# Intake of carbohydrates and SFA and risk of CHD in middle-age adults: the Hordaland Health Study (HUSK)

**DOI:** 10.1017/S1368980020003043

**Published:** 2022-03

**Authors:** Teresa R Haugsgjerd, Grace M Egeland, Ottar K Nygård, Jannicke Igland, Gerhard Sulo, Vegard Lysne, Kathrine J Vinknes, Kjetil Bjornevik, Grethe S Tell

**Affiliations:** 1 Department of Global Public Health and Primary Care, University of Bergen, Årstadveien 17, 5009 Bergen, Norway; 2 Health Registries, Research and Development, The Norwegian Institute of Public Health, Bergen, Norway; 3 Department of Heart Disease, Haukeland University Hospital, Bergen, Norway; 4 Department of Clinical Science, Centre for Nutrition, University of Bergen, Bergen, Norway; 5 Centre for Disease Burden, Norwegian Institute of Public Health, Bergen, Norway; 6 Oral Health Centre of Expertise in Western Norway, Bergen, Norway; 7 Faculty of Medicine, Institute of Basic Medical Sciences, University of Oslo, Oslo, Norway; 8 Department of Nutrition, Harvard T.H. Chan School of Public Health, Boston, MA, USA; 9 Division of Mental and Physical Health, Norwegian Institute of Public Health, Bergen, Norway

**Keywords:** Cohort, SFA, Carbohydrates, CHD

## Abstract

**Objective:**

Limiting SFA intake may minimise the risk of CHD. However, such reduction often leads to increased intake of carbohydrates. We aimed to evaluate associations and the interplay of carbohydrate and SFA intake on CHD risk.

**Design:**

Prospective cohort study.

**Setting:**

We followed participants in the Hordaland Health Study, Norway from 1997–1999 through 2009. Information on carbohydrate and SFA intake was obtained from a FFQ and analysed as continuous and categorical (quartiles) variables. Multivariable Cox regression estimated hazard ratios (HR) and 95 % CI. Theoretical substitution analyses modelled the substitution of carbohydrates with other nutrients. CHD was defined as fatal or non-fatal CHD (ICD9 codes 410–414 and ICD10 codes I20–I25).

**Participants:**

2995 men and women, aged 46–49 years.

**Results:**

Adjusting for age, sex, energy intake, physical activity and smoking, SFA was associated with lower risk (HR_Q4 *v*. Q1_ 0·44, 95 % CI 0·26, 0·76, *P*_trend_ = 0·002). For carbohydrates, the opposite pattern was observed (HR_Q4 *v*. Q1_ 2·10, 95 % CI 1·22, 3·63, *P*_trend_ = 0·003). SFA from cheese was associated with lower CHD risk (HR_Q4 *v*. Q1_ 0·44, 95 % CI 0·24, 0·83, *P*_trend_ = 0·006), while there were no associations between SFA from other food items and CHD. A 5 E% substitution of carbohydrates with total fat, but not SFA, was associated with lower CHD risk (HR 0·75, 95 % CI 0·62, 0·90).

**Conclusions:**

Higher intake of predominantly high glycaemic carbohydrates and lower intake of SFA, specifically lower intake from cheese, were associated with higher CHD risk. Substituting carbohydrates with total fat, but not SFA, was associated with significantly lower risk of CHD.

According to Ancel Keys ‘diet-heart’ hypothesis, a habitually high intake of SFA may increase the risk of CHD due to increases in serum total cholesterol (TC)^(1,2)^. Mensink & Katan^(3)^ published a meta-analysis in 1992, including twenty-seven controlled trials, concluding that the most favourable lipoprotein profile for CHD was achieved if SFA were replaced by unsaturated fatty acids, keeping the intake of total fat unchanged.

The discovery of the additional pathways leading from diet to CHD has made the ‘diet-heart’ hypothesis more complex^(4)^. Advice to reduce SFA as a means to prevent CHD may have, indirectly, increased the intake of carbohydrates^(5,6)^. While carbohydrates have been considered a basis of a healthy diet, with grain products at the base of the Food Guide Pyramid^(7)^, a diet rich in added sugars and refined grains promotes visceral adiposity and reduces energy expenditure^(8–10)^, raising concerns of the potential for increased CHD risk. On the other hand, if dietary carbohydrates are replaced by fat, the postprandial rise in blood glucose and insulin decreases while glucagon secretion increases, resulting in lower CHD risk^(11–14)^. However, among adults with obesity, Hall *et al*.^(15)^ found that restriction of dietary fat was associated with a slightly larger body fat loss than restriction of dietary carbohydrates. Also of twenty-nine diets with different macronutrient compositions tested in mice, only high-fat diets led to overconsumption and weight gain^(16)^. A review indicated that greater high glycaemic index carbohydrate intake was associated with a higher risk of CVD compared with SFA intake^(17)^. Further, recent prospective studies and reviews as well as meta-analyses have shown inconclusive associations between self-reported intakes of either SFA or carbohydrates and fatal and non-fatal CHD^(18–23)^.

Given the inconsistencies in the literature, the objective of the current study was to evaluate the associations of carbohydrate and SFA intakes with incident CHD in a sample of middle-age community-dwelling Norwegian adults, where the intake of carbohydrates varied from 21 to 74 energy percentage (E%) with a median intake of 49 E%, and where the intake of SFA varied from 4 to 25 E% with a median intake of 13 E%.

## Participants and methods

### Study population

The current study is a prospective cohort study of participants in the community-based Hordaland Health Study (HUSK) (https://husk-en.w.uib.no/). The recruitment was based on a cohort from 1992 to 1993 (The Hordaland Homocysteine Study). In 1997–1999, all living cohort members born 1950–1951 and residing in the city of Bergen or the neighbouring suburban municipalities were invited to participate in HUSK. The baseline examinations for the current study were conducted during 1997–1999 as a collaboration between the National Health Screening Service (now The Norwegian Institute of Public Health), The University of Bergen and local health services. The participation rate was 77 %. Participants underwent a brief health examination and provided a non-fasting blood sample. Information on lifestyle was collected via self-administered questionnaires. A semi-quantitative FFQ was completed by 87 % of the participants. A total of 3107 participants aged 46–49 years who answered the FFQ were eligible to be included in the current study.

We excluded twenty-two men and five women who reported prior CHD, and four men and nineteen women due to missing information. Further, we excluded twenty-seven men and thirty-five women who reported extreme energy intakes (below the 1st percentile: <4707·8 kJ for men and 2951·8 kJ for women; or above the 99th percentile: >18907·9 kJ for men and >14944·0 kJ for women). The final study population thus included 2995 participants.

### Dietary assessment

Information on food intake was obtained at baseline (1997–1999) using a 169-item past-year semi-quantitative FFQ, a slightly modified version of a previously described FFQ^(24)^. The validity study of the previous version of the FFQ in a younger population found that the Spearman correlation coefficients between intake of SFA and carbohydrates estimated by the FFQ *v*. weighed food records were 0·44 and 0·57, respectively^(24)^. The FFQ was handed out on the day of the health examination, filled out at home and returned by mail to the HUSK project centre. It includes frequency alternatives (from once a month to several times/d), the number of units eaten and portion sizes (e.g., slices, glasses and spoons) to capture the habitual diet during the past year. The information is presented as individual food or beverage items, food groups and nutrients. Daily nutrient intakes were computed from a database and software system developed at the Department of Nutrition, University of Oslo (KBS, version 3 . 2). The nutrient database is primarily based on the official Norwegian food composition table^(25)^. During dietary data collection in 1997–1999, margarine was undergoing rapid compositional changes where large amounts of *trans*-fatty acids, an important contributor to unsaturated fat^(5,26)^, were being reduced due to legislation in Norway^(26)^. Further, prospectively, there were other changes to unsaturated fat sources^(5)^; thus, unsaturated fat was not evaluated as a primary dietary exposure in the current study.

Measurements used as independent variables in the current study are the total dietary amount of SFA and carbohydrates, as well as intake of SFA and carbohydrates from different food items. All are expressed as E%.

### Health examination and health habits

Baseline examinations included measurements of height, weight, waist circumference, blood pressure and non-fasting blood samples. After at least 2 min seated rest, systolic blood pressure and diastolic blood pressure were measured three times (Dinamap 845 XT equipment (Criticon)). Serum samples of TC, HDL-cholesterol, TAG and glucose were analysed within 7 d at the Department of Clinical Chemistry, Ullevål University Hospital, Oslo, using enzymatic methods with reagents from Boehringer Mannheim (Roche). The Friedewald equation was used for the calculation of LDL-cholesterol. Information on educational level and medication use was self-reported.

Hypertension was considered present if the mean of at least two consecutive measurements of systolic blood pressure was ≥140 mmHg or diastolic blood pressure ≥ 90 mmHg or if the use of medication for hypertension was self-reported.

Participants taking diabetes medications or reported having been diagnosed with diabetes were defined as having diabetes. Also, participants with a serum glucose level >7 mmol/l who had not eaten a meal during the last 8 h, or with a glucose level >11·1 mmol/l and <8 h since their last meal, were defined as having diabetes. Pre-diabetes was defined as having glucose levels between 5·6 and 7 mmol/l at least 8 h after their last meal or between 7·8 and 11 mmol/l <8 h after their last meal.

Participants answered one question on past-year vigorous physical activity resulting in sweating or shortness of breath (none, <1, 1–2 or ≥3 h/week). This variable was treated as a categorical variable with none as the reference.

Participants were classified as either non-smokers, former smokers or current smokers with non-smokers as the reference.

### Outcome

The study endpoints were incident (first time) hospitalisation with CHD (ICD9 codes 410–414 and ICD10 codes I20–I25) as primary or secondary diagnosis or death with CHD as the underlying cause of death. Participants were followed from baseline through 31 December 2009 for CHD events through the Cardiovascular Disease in Norway project (CVDNOR, http://www.cvdnor.no)^(27,28)^ and The Cause of Death Registry. There were 107 non-fatal and five fatal episodes. Follow-up time was calculated as time from participation until CHD, death from other causes, emigration or 31 December ·2009, whichever occurred first.

### Statistical analyses

Descriptive characteristics include numbers with percentages and medians (25th, 75th percentiles) for categorical and continuous variables, respectively. Spearman’s rank correlation (rho, *ρ*) was used to evaluate correlations between quartiles of carbohydrate intake and SFA intake with baseline characteristics. In addition, Spearman correlations of intake of carbohydrates with total fat and SFA were evaluated. To evaluate linear trends in baseline characteristics across quartiles of carbohydrate and SFA intakes as percentage of total energy intake, we used ordinal logistic regression for categorical outcome variables, logistic regression for dichotomous outcome variables and linear regression for continuous outcome variables where median intake as E% within each quartile group was used as the independent variable in the analyses. Cox proportional hazard models were used to calculate adjusted hazard ratios (HR) with 95 % CI for CHD associated with continuous and quartile intake of carbohydrates and SFA. The included covariates were potential confounders associated with the intake of carbohydrates and SFA and with CHD, which also modified the association of either SFA or carbohydrate with CHD when included in the multivariable model. Two primary analyses are presented: model 1 adjusted for age (continuous (years)), sex and total energy intake (continuous (kcal/d)); model 2 additionally adjusted for vigorous physical activity (none *v*. <1 h/week, 1–2 h/week or ≥3 h/week) with none as the reference and smoking habits (non-smokers *v*. previous smokers and non-smokers *v*. current smokers). The following additional confounders were also evaluated, but inclusion of the variables did not materially alter the associations of SFA or carbohydrates with CHD: family history of myocardial infarction, educational level and alcohol intake (E%). Further, carbohydrate analyses also evaluated consistency in results after adjustment for energy-adjusted fibre from bread, fruit and vegetables. SFA analyses were further adjusted for energy-adjusted intake of cholesterol, PUFA and protein.

Supplementary analyses evaluated models adjusted for age, sex and energy intake (model 1), with additional adjustments for HDL-cholesterol, LDL-cholesterol, TAG, glucose, systolic blood pressure, diastolic blood pressure and BMI (model 2); with additional adjustments for diabetes/prediabetes, hypertension, family history of myocardial infarction, statins, oral hypoglycaemics (including metformin) and insulin and anti-hypertensive medications (model 3) and with additional adjustments for smoking, physical activity, alcohol consumption in E% and education (model 4) (see online supplementary material, Supplemental Table S1). To test for linear trends across intake quartiles, median intake as E% within each quartile group was used as the independent variable. We also evaluated SFA from cheese and SFA when excluding the contribution from cheese for their associations with incident CHD. In additional supplementary analyses, we stratified intake of SFA on smoking habits (see online supplementary material, Supplemental Table S2) and we also evaluated associations between carbohydrates and SFA from other specific food groups and CHD risk (see online supplementary material, Supplemental Tables S3 and S4). Missing data were handled with listwise deletion.

The proportional hazard assumption was evaluated using Schoenfeld’s test.

To evaluate the continuous association between exposure and outcome, and assess potential non-linear associations, smoothed penalised splines were plotted^(29)^.

We used theoretical substitution analyses to model the substitution of carbohydrates with SFA^(30)^. Variables for the E% (per 5 E% unit increments) of all macronutrients except carbohydrates (SFA, monounsaturated fat, PUFA, protein and alcohol) were included in a Cox model with adjustment for total energy intake, age, sex, physical activity and smoking habits. The HR for SFA is then interpreted as the change in estimated risk for each 5 E% unit increase in SFA while holding all other variables in the model constant but allowing for concomitant decreases in carbohydrate intake as all sources of macronutrients sum to 100 % of energy intake. The same approach was used to evaluate the theoretical substitution of carbohydrates with other macronutrients: total fat, protein and PUFA intake per 5 E% unit increase in a model with other macronutrients except carbohydrates^(30)^.

Sensitivity analyses were conducted where we excluded the first 2 years of observation following the baseline assessment in all of the above analyses.

Statistical analyses were performed using Stata version 15 (StataCorp LP) and R version 3.4.0 (https://www.r-project.org/), The R Foundation for Statistical Computing. *P*-values <0·05 were considered statistically significant.

## Results

### Characteristics of the study population

At baseline, mean age was 48 (sd 0·7) years, median BMI was 24·9 (25th, 75th percentiles 22·8, 27·4) kg/m^2^, 33·5 % smoked daily, 45·9 % reported at least 1 h vigorous physical activity per week and 25·3 % had indications of reduced metabolic health defined as having hypertension, pre-diabetes or diabetes. Intake of total fat ranged from 14 to 53 E% with a median intake of 33 E%. Intake of total carbohydrates ranged from 21 to 74 E% with a median intake of 49 E%. Less than 1 and 6 % had an intake of carbohydrates at or below 30 and 40 E%, respectively, while 3 and <1 % had an intake of carbohydrates at or above 60 and 70 E%, respectively. Intake of protein ranged from 6 to 30 E% with a median intake of 16 E%, while intake of SFA ranged from 4 to 25 E% with a median intake of 13 E%. Less than 1 % had an intake of SFA at or below 6 E%, while 14 % had an intake at or above 15 E%.

During a mean 10·8 (sd 1·3) years of follow-up, representing 32 449 person-years among 2995 participants (1282 men and 1713 women), we documented 112 incident CHD events. Due to missing values (2·1 % for smoking habits and 3·8 % for physical activity), multivariable-adjusted analyses included 2820 participants (1224 men and 1596 women) and 105 CHD events. Sixty participants died due to other causes during follow-up. When evaluating Spearman correlations between carbohydrate quartiles and baseline characteristics, all correlations (*ρ*) were between −0·2 and <0·1. However, evaluation of baseline characteristics by quartiles of carbohydrate intake identified that the proportion of participants performing at least 1 h vigorous physical activity per week was higher in higher quartiles, while the proportions of men, daily smokers and participants with glucose intolerance were lower in higher quartiles (Table 1). Also, waist circumference, serum levels of TC, LDL-cholesterol and HDL-cholesterol were lower in higher carbohydrate quartile groups. Intakes of total fat, protein and alcohol were lower with higher quartiles of carbohydrate intake. Bread was the major contributor to carbohydrates in this population. While intake (g/d per 1000 kcal) of bread, sweetened beverages, juice, and fruit and berries (both fresh and canned/preserved) was higher with higher quartiles of carbohydrate intake, there were less noticeable differences for other carbohydrate sources across quartiles. Vegetable and fibre intakes (g/d per 1000 kcal), for example, were similar in the various carbohydrate intake quartiles (Table 1).

**Table 1:** Baseline characteristics by quartiles of carbohydrate intake (energy percentage (E%)), The Hordaland Health Study*

	Total		Q1		Q2		Q3		Q4		
	Median/*n*	25th, 75th percentiles/%	Median/*n*	25th, 75th percentiles/%	Median/*n*	25th, 75th percentiles/%	Median/*n*	25th, 75th percentiles/%	Median/*n*	25th, 75th percentiles/%	*P*_trend_ †
Carbohydrates (E%)	49	46, 53	43	40, 44	48	47, 48	51	50, 52	56	54, 58	<0·001
Carbohydrates (g/d per 1000 kcal)	123	114, 132	107	101, 110	119	116, 121	127	125, 129	139	135, 145	<0·001
Participants (*n*)	2995		749		749		748		749		
Age (years)	48	47, 48	48	47, 48	48	47, 48	48	47, 48	48	47, 48	0·822
Men	1282	42·8	348	46·5	332	44·3	320	42·8	282	37·7	0·001
College and/or university education	1138	38·3	253	34·0	308	41·3	286	38·4	291	39·5	0·059
Family history of infarction	1186	40·9	301	42·0	283	39·0	297	41·0	305	41·7	0·952
Smoking habits											<0·001
Previous smokers	915	31·2	218	29·5	225	30·4	240	32·9	232	32·1	
Current smokers	982	33·5	337	45·7	268	36·2	203	27·8	174	24·1	
Physical activity											<0·001
None	745	25·9	218	30·0	188	26·0	174	24·1	165	23·3	
<1 h/week	813	28·2	220	30·2	225	31·2	189	26·2	179	25·3	
1–2 h/week	908	31·5	189	26·0	232	32·1	246	34·1	241	34·0	
≥3 h/week	415	14·4	101	13·9	77	10·7	113	15·7	124	17·5	
Hypertension	708	23·7	171	22·9	184	24·6	178	23·8	175	23·4	0·886
Glucose intolerance											0·050
Pre-diabetes	66	2·2	21	2·8	19	2·6	14	1·9	12	1·6	
Diabetes	27	0·9	9	1·2	8	1·1	3	0·4	7	0·9	
Casual glucose (mmol/l)‡	5·01	4·64, 5·49	5·04	4·69, 5·50	5·02	4·65, 5·53	5·01	4·64, 5·45	4·96	4·60, 5·44	0·094
BMI (kg/m^2^)	24·9	22·8, 27·4	25·0	23·0, 27·5	24·7	22·7, 27·2	24·9	22·7, 27·4	24·9	22·8, 27·4	0·410
Waist circumference (cm)	85·0	77·0, 94·0	86·0	77·0, 95·0	85·0	76·0, 93·0	85·0	77·0, 93·0	84·0	76·0, 93·0	0·003
Cholesterol (mmol/l)‡	5·65	5·06, 6·30	5·72	5·13, 6·45	5·66	5·04, 6·28	5·64	5·04, 6·27	5·57	5·01, 6·22	0·001
LDL-cholesterol (mmol/l)‡	3·57	3·01, 4·17	3·60	3·03, 4·27	3·58	3·01, 4·15	3·57	3·02, 4·18	3·51	2·96, 4·09	0·010
HDL-cholesterol (mmol/l)‡	1·28	1·06, 1·53	1·29	1·08, 1·54	1·29	1·05, 1·55	1·28	1·06, 1·53	1·26	1·03, 1·51	0·017
TAG (mmol/l)‡	1·40	1·01, 2·04	1·42	1·01, 2·1	1·39	1·00, 1·94	1·37	1·00, 2·01	1·41	1·02, 2·10	0·933
Medications for											
Diabetes	14	0·5	4	0·5	4	0·5	2	0·3	4	0·5	0·842
Hypertension	134	4·5	29	3·9	40	5·3	33	4·4	32	4·3	0·879
Hypercholesterolemia	50	1·7	19	2·5	7	0·9	12	1·6	12	1·6	0·262
Dietary intake											
SFA (E%)	13	11, 14	14	13, 16	13	12, 14	12	11, 13	11	9, 12	<0·001
Total fat (E%)	33	29, 36	38	35, 41	34	32, 36	31	30, 33	27	25, 29	<0·001
Protein (E%)	16	14, 17	17	15, 18	16	15, 17	16	14, 17	15	14, 16	<0·001
Alcohol (E%)	1	0, 3	2	1, 4	1	0, 3	1	0, 2	1	0, 2	<0·001
Cholesterol (mg/d per 1000 kcal)	130	110, 152	152	131, 176	137	119, 156	126	111, 142	107	91, 125	<0·001
Fibre (g/d per 1000 kcal)	11	10, 13	10	9, 11	11	10, 12	12	10, 14	13	11, 16	<0·001
Energy intake (kcal§/d)	2057	1690, 2550	2133	1751, 2654	2128	1750, 2578	2079	1687, 2561	1917	1559, 2399	<0·001
Intake of food items											
Cakes											
g/d per 1000 kcal	10	5, 17	8	3, 14	11	5, 18	11	6, 18	10	5, 16	0·001
E% carbohydrates	1·5	0·7, 2·6	1·2	0·5, 2·1	1·6	0·7, 2·6	1·6	0·9, 2·8	1·5	0·7, 2·6	<0·001
Snacks											
g/d per 1000 kcal	2	1, 5	3	1, 6	2	1, 5	2	1, 4	1	0, 4	<0·001
E% carbohydrates	0·3	0·1, 0·7	0·4	0·1, 0·8	0·3	0·1, 0·7	0·3	0·1, 0·7	0·2	0, 0·6	<0·001
Soft drinks with sugar											
g/d per 1000 kcal	25	0, 65	18	0, 43	28	2, 68	27	2, 63	34	1, 86	<0·001
E% carbohydrates	1·0	0, 2·6	0·7	0, 1·7	1·1	0·1, 2·8	1·1	0·1, 2·6	1·4	0·0, 3·6	<0·001
Fresh fruit and berries											
g/d per 1000 kcal	62	35, 98	43	24, 71	59	35, 91	66	42, 104	85	48, 139	<0·001
E% carbohydrates	3·3	1·9, 5·3	2·3	1·3, 3·7	3·2	1·9, 4·8	3·6	2·2, 5·6	4·5	2·6, 7·5	<0·001
Juice											
g/d per 1000 kcal	20	2, 49	12	0, 32	21	4, 48	26	6, 58	23	2, 63	<0·001
E% carbohydrates	0·8	0·1, 2·0	0·5	0, 1·3	0·8	0·2, 1·9	1·0	0·2, 2·3	0·9	0·1, 2·5	<0·001
Conserved fruit and berries											
g/d per 1000 kcal	13	3, 25	7	1, 18	13	4, 24	16	6, 28	17	5, 32	<0·001
E% carbohydrates	2·3	0·4, 4·8	1·0	0, 3·4	2·3	0·5, 4·4	2·9	0·9, 5·3	3·1	0·7, 6·1	<0·001
Bread											
g/d per 1000 kcal	85	69, 105	77	60, 93	83	67, 99	88	72, 108	98	78, 120	<0·001
E% carbohydrates	16·4	13·2, 20·1	14·7	11·7, 17·8	15·8	13·0, 18·9	16·8	13·8, 20·7	18·7	14·8, 22·9	<0·001
Rice, pasta, flour, cereals											
g/d per 1000 kcal	19	12, 28	16	10, 23	19	12, 26	20	13, 29	21	13, 33	<0·001
E% carbohydrates	3·7	2·3, 5·9	3·0	2·0, 4·6	3·6	2·3, 5·5	4·1	2·6, 6·4	4·5	2·6, 7·3	<0·001
Potatoes											
g/d per 1000 kcal	48	32, 67	47	30, 63	47	33, 64	48	31, 67	52	33, 71	<0·001
E% carbohydrates	4·1	2·7, 5·6	4·0	2·6, 5·4	4·1	2·9, 5·5	4·1	2·7, 5·6	4·3	2·8, 5·8	<0·001
Vegetables											
g/d per 1000 kcal	85	54, 131	82	53, 122	85	55, 129	88	57, 132	87	52, 136	0·136
E% carbohydrates	2·2	1·5, 3·2	2·2	1·4, 3·1	2·2	1·5, 3·2	2·3	1·5, 3·2	2·3	1·4, 3·3	0·038

Q, quartile.

* Values are presented as *n* and % and median (25th, 75th percentiles) for categorical and continuous variables, respectively.

† Logistic regression for categorical variables with two categories, ordered logistic regression when more than two categories and linear regression for continuous variables where median intake as E% within each quartile group was used as the independent variable in the analyses.

‡ In serum.

§ To convert kcal to kJ, multiply by 4·184.

When evaluating Spearman correlations between SFA quartiles and the baseline characteristics, all rhos (*ρ*) were between –0·1 and <0·1. However, the percentage daily smokers were higher with higher quartiles of SFA intake, while the percentage of participants who were men, performed at least 1 h vigorous physical activity per week or had hypertension was lower with higher quartiles (Table 2). Also, BMI, waist circumference and TAG levels, as well as the percentage taking medications for hypercholesterolaemia, were lower with higher SFA quartiles. While intake of cheese was higher with higher quartiles of SFA intake, there were less noticeable differences for other SFA sources across quartiles. Family history of myocardial infarction did not differ between quartiles of carbohydrate (*P*_trend_ 0·95) or SFA (*P*_trend_ 0·23) intake as percentage of total energy.

**Table 2:** Baseline characteristics by quartiles of saturated fat intake (energy percentage (E%)), The Hordaland Health Study*

	Total		Q1		Q2		Q3		Q4		*P*_trend_ †
	Median/*n*	25th, 75th percentiles/%	Median/*n*	25th, 75th percentiles/%	Median/*n*	25th, 75th percentiles/%	Median/*n*	25th, 75th percentiles/%	Median/*n*	25th, 75th percentiles/%	
SFA (E%)	13	11, 14	10	9, 11	12	11, 12	13	13, 14	15	15, 16	<0·001
SFA (g/d per 1000 kcal)	14	12, 16	11	10, 12	13	13, 14	15	14, 15	17	16, 18	<0·001
Participants (*n*)	2995		749		749		748		749		
Age (years)	48	47, 48	48	48, 49	48	47, 48	48	47, 48	48	47, 48	0·151
Men	1282	42·8	327	43·7	349	46·6	318	42·5	288	38·5	0·016
College and/or university education	1138	38·3	291	39·4	281	37·6	290	39·2	276	37·0	0·121
Family history of infarction	1186	40·9	316	43·4	295	40·7	284	39·2	291	40·5	0·227
Smoking habits											<0·001
Previous smoker	915	31·2	248	34·2	241	32·9	230	31·2	196	26·7	
Current smoker	982	33·5	197	27·2	233	31·8	259	35·1	293	39·9	
Physical activity											<0·001
None	745	25·9	150	21·0	196	27·3	199	27·4	200	27·7	
<1 h/week	813	28·2	192	26·9	168	23·4	221	30·4	232	32·2	
1–2 h/week	908	31·5	241	33·7	243	33·8	221	30·4	203	28·2	
≥3 h/week	415	14·4	132	18·5	112	15·6	85	11·7	86	11·9	
Hypertension	708	23·7	196	26·2	183	24·4	179	24·0	150	20·0	0·006
Glucose intolerance											0·296
Pre-diabetes	66	2·2	15	2·0	17	2·3	11	1·5	23	3·1	
Diabetes	27	0·9	6	0·8	6	0·8	9	1·2	6	0·8	
Casual glucose (mmol/l)‡	5·01	4·64, 5·49	5·04	4·66, 5·51	5·01	4·66, 5·47	4·99	4·65, 5·49	4·98	4·60, 5·43	0·400
BMI (kg/m^2^)	24·9	22·8, 27·4	25·2	23·0, 27·6	25·2	23·0, 27·5	24·8	23·0, 27·4	24·6	22·2, 27·1	0·002
Waist circumference (cm)	85·0	77·0, 94·0	85·0	77·0, 94·0	86·0	78·0, 94·0	85·0	77·0, 93·0	84·0	75·0, 93·0	0·021
Cholesterol (mmol/l)‡	5·65	5·06, 6·30	5·64	5·06, 6·35	5·61	5·01, 6·26	5·69	5·10, 6·28	5·65	5·08, 6·35	0·729
LDL-cholesterol (mmol/l)‡	3·57	3·01, 4·17	3·55	2·96, 4·17	3·53	2·99, 4·11	3·58	3·07, 4·17	3·61	3·02, 4·23	0·250
HDL-cholesterol (mmol/l ‡	1·28	1·06, 1·53	1·29	1·06, 1·53	1·25	1·04, 1·50	1·29	1·07, 1·54	1·32	1·08, 1·57	0·064
TAG (mmol/l)‡	1·40	1·01, 2·04	1·41	1·04, 2·15	1·47	1·03, 2·08	1·40	1·01, 1·99	1·33	0·95, 1·93	0·005
Medications for											
Diabetes	14	0·5	3	0·4	2	0·3	6	0·8	3	0·4	0·704
Hypertension	134	4·5	36	4·8	37	4·9	33	4·4	28	3·7	0·270
Hypercholesterolaemia	50	1·7	20	2·7	11	1·5	14	1·9	5	0·7	0·007
Dietary intake											
Carbohydrates (E%)	49	46, 53	54	51, 58	51	48, 53	48	45, 51	45	42, 48	<0·001
Total fat (E%)	33	29, 36	27	25, 29	31	30, 33	34	32, 36	37	35, 40	<0·001
Protein (E%)	16	14, 17	16	14, 17	16	14, 17	16	15, 17	16	14, 17	0·876
Alcohol (E%)	1	0, 3	1	0, 3	1	0, 3	1	0, 3	1	0, 2	<0·001
Cholesterol (mg/d per 1000 kcal)	130	110, 152	115	95, 137	128	110, 149	133	116, 156	143	122, 164	<0·001
Fibre (g/d per 1000 kcal)	11	10, 13	13	11, 16	12	10, 13	11	10, 13	10	8, 11	<0·001
Energy intake (kcal§/d)	2057	1690, 2550	1900	1537, 2335	2093	1731, 2584	2112	1712, 2623	2182	1773, 2584	<0·001
Intake of food items											
Butter||											
g/d per 1000 kcal	0	1	0	0	0	1	0	1	0	3	<0·001
E% SFA	0	1	0	0	0	0	0	0	0	1	<0·001
Cheese											
g/d per 1000 kcal	13	7, 21	9	4, 14	11	7, 18	15	9, 24	19	11, 32	<0·001
E% SFA	2	1, 3	1	1, 2	2	1, 2	2	1, 3	3	2, 4	<0·001
Margarine											
g/d per 1000 kcal	3	2, 8	2	1, 4	3	2, 7	3	2, 9	3	2, 11	<0·001
E% SFA	1	1, 2	1	0, 1	1	1, 2	1	1, 2	1	1, 3	<0·001
Milk and milk products											
g/d per 1000 kcal	129	65, 205	131	61, 214	140	71, 213	128	69, 196	119	61, 196	0·031
E% SFA	1	1, 2	1	1, 2	1	1, 2	1	1, 2	2	1, 3	<0·001
Meat and meat products											
g/d per 1000 kcal	55	41, 70	49	36, 63	56	43, 70	57	43, 74	57	42, 73	<0·001
E% SFA	2	2, 3	2	2, 3	2	2, 3	2	2, 3	3	2, 3	<0·001
Minced meat products											
g/d per 1000 kcal	24	15, 34	20	12, 29	25	16, 33	26	17, 36	26	17, 35	<0·001
E% SFA	1	1, 2	1	1, 1	1	1, 2	1	1, 2	1	1, 2	<0·001

Q, quartile.

* Values are presented as *n* and % and median and 25th, 75th percentiles for categorical and continuous variables, respectively.

† Logistic regression for categorical variables with two categories, ordered logistic regression when more than two categories and linear regression for continuous variables where median intake as E% within each quartile group was used as the independent variable in the analyses.

‡ In serum.

§ To convert kcal to kJ, multiply by 4·184.

|| Intake is reported as median and mean because of a large number of zero intake reporting.

### Associations between intake of carbohydrates and SFA and incident CHD

Higher intake of carbohydrates was borderline significantly associated with higher risk of CHD in model 1 (adjusted for age, sex and energy intake) (HR_Q(quartile)4 *v*. Q1_ 1·63, 95 % CI 0·96, 2·76, *P*_trend_ = 0·056) (Table 3). This association became stronger and significant after further adjustment for smoking habits and physical activity (model 2) (HR_Q4 *v*. Q1_ 2·10, 95 % CI 1·22, 3·63, *P*_trend_ = 0·003). Also, continuous analyses (per 2 E%) showed significantly higher risk of CHD with higher intake of carbohydrates (HR 1·12, 95 % CI 1·05, 1·20), after adjusting for age, sex, energy intake, smoking habits and physical activity. Further adjustments for intermediate factors, relevant medications and potential confounders did not materially influence the association (see online supplementary material, Supplemental Table S1).

**Table 3:** Associations between macronutrients and risk of incident CHD, The Hordaland Health Study. Mean follow-up time 10·8 years

Intake of macronutrients (range in E%)*	*n*	CHD		Model 1†		Model 2‡	
		*n*	%	HR	95 % CI	HR	95 % CI
Carbohydrates	2995	112					
Q1 (21, 45)	749	23	3·1	1 (ref)		1 (ref)	
Q2 (45, 49)	749	25	3·3	1·11	0·63, 1·96	1·09	0·60, 1·98
Q3 (49, 53)	748	29	3·9	1·30	0·75, 2·24	1·67	0·96, 2·89
Q4 (53, 74)	749	35	4·7	1·63	0·96, 2·76	2·10	1·22, 3·63
*P*_trend_ §				0·056		0·003	
Continuous (per 2 E%)	2995	112		1·08	1·01, 1·15	1·12	1·05, 1·20
Saturated fat	2995	112					
Q1 (4, 11)	749	44	5·9	1 (ref)		1 (ref)	
Q2 (11, 13)	749	24	3·2	0·55	0·34, 0·91	0·46	0·27, 0·78
Q3 (13, 14)	748	23	3·1	0·55	0·33, 0·92	0·47	0·28, 0·79
Q4 (14, 25)	749	21	2·8	0·53	0·32, 0·90	0·44	0·26, 0·76
*P*_trend_ §				0·013		0·002	
Continuous (per 2 E%)	2995	112		0·82	0·70, 0·97	0·78	0·66, 0·92
Saturated fat from cheese	2995	112					
Q1 (0, 1)	749	45	6·0	1 (ref)		1 (ref)	
Q2 (1, 2)	748	28	3·7	0·68	0·43, 1·10	0·78	0·49, 1·27
Q3 (2, 3)	749	25	3·3	0·64	0·39, 1·04	0·60	0·35, 1·03
Q4 (3, 18)	749	14	1·9	0·38	0·21, 0·70	0·44	0·24, 0·83
*P*_trend_ §				0·002		0·006	
Continuous (per 1 E%)	2995	112		0·84	0·74, 0·97	0·87	0·75, 0·99
Saturated fat after excluding saturated fat from cheese	2995	112					
Q1 (3, 9)	748	34	4·6	1 (ref)		1 (ref)	
Q2 (9, 10)	750	29	3·9	0·85	0·51, 1·39	0·71	0·42, 1·20
Q3 (10, 12)	748	20	2·7	0·56	0·32, 0·98	0·46	0·26, 0·81
Q4 (12, 21)	749	29	3·9	0·81	0·49, 1·34	0·58	0·34, 0·98
*P*_trend_ §				0·277		0·030	
Continuous (per 2 E%)	2995	112		0·94	0·79, 1·12	0·85	0·71, 1·02

E%, energy percentage; CHD, incident CHD; *n*, number of participants; HR, hazard ratio; Q, quartile.

* Minimum and maximum intake of the macronutrient.

† Cox proportional hazard regression analysis adjusted for age, sex and energy intake.

‡ Adjusted in addition for physical activity and smoking habits.

§ *P*_trend_, to test for linear trends across quartiles, we modelled the median intake of each quartile as a continuous variable.

**Fig. 1:**
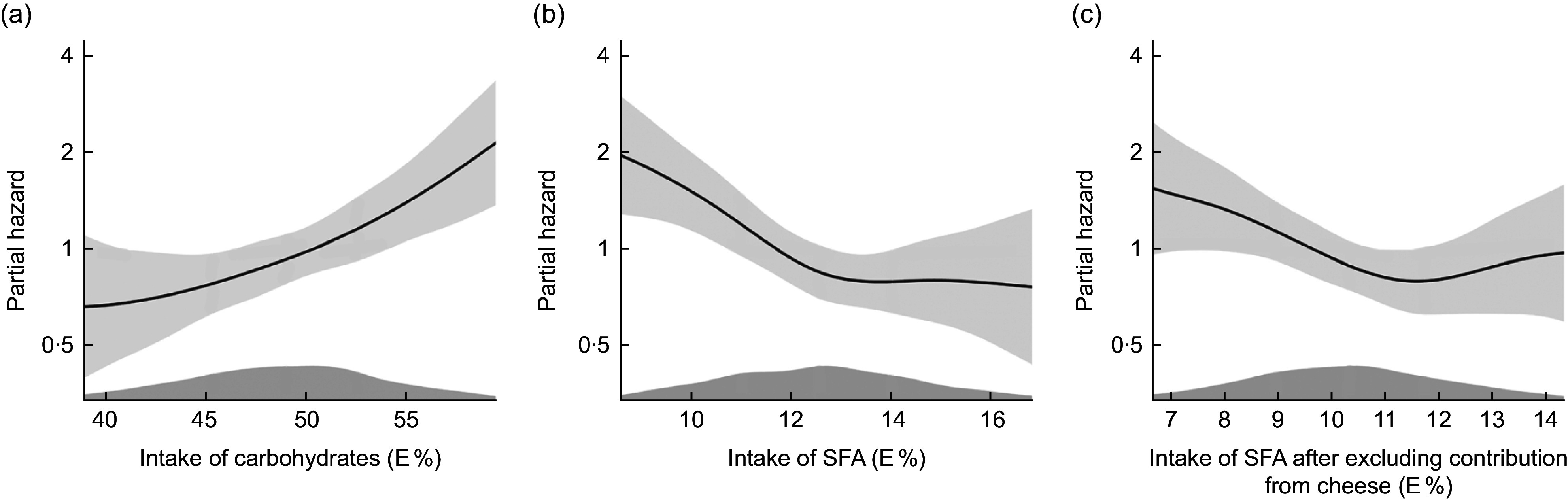
Cox proportional hazards regression with penalized splines, The Hordaland Health Study. Distribution of partial hazard (black line) with 95% CI (shadow) for CHD across the distribution of a) intake of carbohydrates in E%, b) intake of saturated fatty acids (SFA) in E% and c) intake of SFA after excluding contribution from cheese in E%. The model includes age, sex, energy intake, physical activity and smoking habits. Intake above the 5^th^th percentile and below the 95^th^ percentile is included in the figure

Plotting the data adjusting for model 2 covariates indicated a linear relationship (Fig. 1(a)).

When examining the association between carbohydrates from various food items, we found no associations with risk of CHD (see online supplementary material, Supplemental Table S3).

A high intake of SFA was significantly associated with lower risk of CHD in the model adjusted for age, sex and energy intake (model 1) (HR_Q4 *v*. Q1_ 0·53, 95 % CI 0·32, 0·90, *P*_trend_ = 0·013) (Table 3). This association became stronger after further adjustment for smoking habits and physical activity (model 2) (HR_Q4 *v*. Q1_ 0·44, 95 % CI 0·26, 0·76, *P*_trend_ = 0·002). Also, continuous analyses (per 2 E%) showed significantly lower risk of CHD with higher intake of SFA (HR 0·78, 95 % CI 0·66, 0·92), after adjusting for age, sex, energy intake, smoking habits and physical activity. Further adjustments for intermediate factors, relevant medications and potential confounders did not materially influence the association (see online supplementary material, Supplemental Table S1).

Figure 1(b) illustrates lower risk of CHD with a higher intake of SFA until an intake of about 13 E%, after which the curve levelled off, after adjustment for model 2 variables.

When stratifying on smoking habits, there was a tendency of lower risk of CHD with higher intake of SFA in all groups, but less so among current smokers (see online supplementary material, Supplemental Table S2).

When examining the association between SFA from various food items, we found that only SFA from cheese was significantly associated with a lower risk of CHD (Table 3). The median intake of SFA from cheese ranged from 0·5 E% (Q1) to 4·1 E% (Q4). After adjustments for age, sex, energy intake, physical activity and smoking habits (model 2), SFA from cheese was significantly associated with lower risk of CHD in the quartile analyses (HR_Q4 *v*. Q1_ 0·44, 95 % CI 0·24, 0·83, *P*_trend_ = 0·006). Results from the evaluation of SFA from cheese as a continuous variable (per 1 E%) were similar.

We further evaluated the association between SFA and CHD after excluding the SFA contribution from cheese, and in the quartile analyses, we found that intake of SFA after exclusion of cheese was associated with lower risk of CHD (HR_Q4 *v*. Q1_ 0·58, 95 % CI 0·34, 0·98, *P*_trend_ = 0·030), after adjustment for age, sex, energy intake, physical activity and smoking habits (model 2, Table 3). Results from the continuous analyses were in the same direction as the quartile analyses. Upon further evaluation (Fig. 1(c)), we observed deviations from linearity in the association between SFA intake and CHD risk after excluding SFA from cheese.

**Fig. 2:**
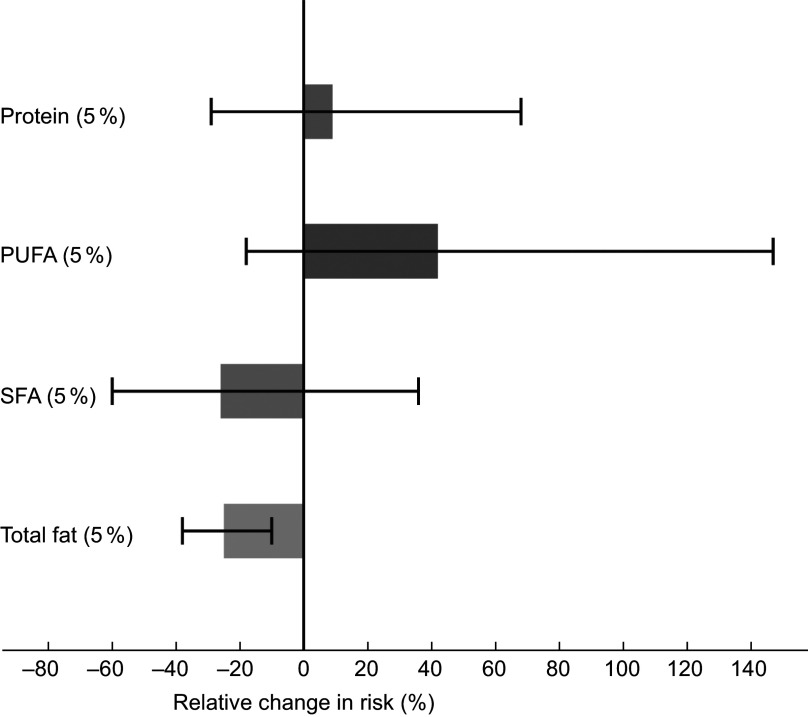
Theoretical substitution analyses, illustrating an isocaloric substitution of 5E% from carbohydrates with total fat, saturated fatty acids (SFA), polyunsaturated fatty acids (PUFA) or protein and its association with CHD. Adjusted for age, sex, energy intake, physical activity and smoking habits. Mean 10·8 years follow-up of the Hordaland Health Study participants

Higher intake of total carbohydrates correlated significantly with lower intake of SFA (*ρ* = −0·6, *P* < 0·001) and lower intake of total fat (*ρ* = −0·8, *P* < 0·001). Results from the theoretical substitution analyses are shown in Fig. 2. Substitution of 5 % of total energy intake from carbohydrates with SFA was associated with a 26 % lower risk of CHD (model 2 HR 0·74, 95 % CI 0·40, 1·36), although not statistically significant. A substitution of carbohydrates with total fat was also associated with lower risk of CHD (model 2 HR 0·75, 95 % CI 0·62, 0·90). To further evaluate whether substitution analyses of carbohydrates with SFA or with total fat could be attributed to an underlying beneficial effect of PUFA, we evaluated results of analyses substituting carbohydrates with PUFA in which we found a non-significant association with incident CHD (model 2 HR 1·42, 95 % CI 0·82, 2·47). Further, the substitution of carbohydrates with protein was not associated with the risk of CHD (model 2 HR 1·09, 95 % CI 0·71, 1·68). When adjusting for age, sex and energy intake only, results of all substitution models were in the same direction as in the fully adjusted model, but were non-significant.

Exclusion of events occurring during the first 2 years of follow-up yielded no material differences in results.

## Discussion

In this community-based study population, high intake of carbohydrates and low intake of SFA were associated with higher risk of incident CHD. Intake of SFA from cheese was significantly associated with lower CHD risk. When evaluating SFA intake after excluding the contribution of SFA from cheese, the association became weaker, but remained significant. Substituting 5 % of total energy intake from carbohydrates with SFA and total fat was associated with lower CHD risk (HR of 0·74 and 0·75, respectively), but was statistically significant only for total fat. The lack of a statistically significant finding for SFA may reflect, in part, the narrower range of SFA intake compared with total fat and carbohydrate intake.

Carbohydrates reflect a variety of sources including sucrose, fructose and refined cereals, as well as fibre-rich whole grains, vegetables and legumes. Refined carbohydrates and added sugar accounted for a large part of carbohydrate intake in the Norwegian diet at the time of HUSK baseline in 1997–1999^(5)^. Even today, few Norwegians comply with the Nordic nutrition recommendations for fibre intake^(31,32)^. Per capita sales data indicate that intake of sugar-containing foods and beverages peaked at the end of the 1990s^(5)^. In addition, a nationwide diet survey among men and women 16–79 years of age (1997–1999) found that their diet contained inadequate amounts of food products rich in fibre and that the intake of added sugar was 10 and 9 E% among men and women, respectively^(33)^. When evaluating baseline characteristics in this cohort, the intake of fruit and berries, sugar-sweetened beverages and juice doubled from the lowest to the highest quartile of carbohydrate intake. In contrast, intake of rice, pasta, flour and cereals was only modestly higher and vegetable intake did not differ across quartiles of total carbohydrate intake. Also, while recommended intake of fibre is ≥25 g/d in women and ≥35 g/d in men^(31)^, the median intake of fibre in the total study population was 24 g/d and the median fibre intake in the highest quartile of carbohydrate intake was 26 g/d. However, FFQ are affected by systematic errors and do not precisely estimate dietary intake; therefore, these data should be interpreted with caution.

Previous cohort studies and meta-analyses have shown diverse results regarding the association between intake of carbohydrates and CHD when evaluating total carbohydrates. A study of men and women 30–59 years of age found that carbohydrate intake was associated with a lower CHD mortality risk (RR 0·96, 95 % CI 0·94, 0·99)^(34)^. However, a large cohort study of individuals aged 35–70 years found that higher carbohydrate intake was not associated with the risk of CVD (HR 1·01, 95 % CI 0·88, 1·15) or myocardial infarction (HR 0·90, 95 % CI 0·73, 1·10)^(23)^. Carbohydrate intake was not consistently associated with CHD when different sources of carbohydrates were considered separately. Li *et al*.^(35)^ found in a cohort study that higher intake of carbohydrates from whole grains was associated with lower risk of incident CHD (HR 0·90, 95 % CI 0·83, 0·98), while carbohydrates from refined starches/added sugars were positively associated with higher risk of CHD (HR 1·10, 95 % CI 1·00, 1·21).

Fung *et al*.^(36)^ studied the association between consumption of sugar-sweetened beverages and the risk of CHD in the Nurses’ Health Study and found that regular consumption of sugar-sweetened beverages was associated with a higher CHD risk. In addition, a meta-analysis of cohort studies reported that intake of sugar-sweetened beverages was associated with increased risk of myocardial infarction^(37)^. In randomised controlled trials, dietary sugar intake has been found to increase blood pressure and serum TAG, TC and LDL-cholesterol^(38)^. While we did not identify any one particular source of carbohydrates to contribute to the overall carbohydrate association with CHD, we did note differences in the quality of carbohydrate sources between low to high carbohydrate intake quartiles where intake of fibre, vegetables and many carbohydrate sources remained essentially stable, while bread, sugar-sweetened beverages, juice, and preserved and fresh fruit and berries increased across the quartiles of carbohydrate intake. Thereby indicating that increased carbohydrate intake in the current study population represented increases in low-fibre and higher sucrose/fructose carbohydrates.

SFA intake in our study population came primarily from dairy products, especially cheese. The intake of cheese more than doubled from the lowest to the highest quartile of SFA intake, and cheese was also the main contributor to SFA intake in the highest quartile. As dairy products are important contributors of SFA, the general recommendation in Norway has been to reduce the intake of high-fat dairy products^(39)^. However, studies do not consistently support that this recommendation would reduce risk of CHD^(40–42)^. A systematic review and meta-analysis did not report a statistically significant association between total dairy intake and CHD (Summary RR 0·91, 95 % CI 0·80, 1·04)^(41)^. Further, the only dairy product significantly associated with lower CHD risk was cheese (Summary RR 0·82, 95 % CI 0·72, 0·93)^(41)^. In addition, Qin *et al*.^(42)^ reported no association between dairy intake and CHD (RR 0·94, 95 % CI 0·82, 1·07), and CHD risk was lowered by cheese consumption also in this study (RR 0·84, 95 % CI 0·71, 1·00).

We found that intake of SFA from cheese was the only food source associated with a lower risk of CHD. Underlying mechanisms for a potential CHD protective effect of cheese may relate to (i) fermentation which may influence dairy fat’s contribution to LDL-cholesterol^(43)^ and (ii) menaquinones (vitamin K_2_) which comes primarily from cheese in European diets^(44)^. Geleijnse *et al*.^(45)^ found that menaquinone intake was inversely associated with serum TC and aortic calcification and positively associated with serum HDL-cholesterol. Menaquinones transported together with SFA may, therefore, be associated with lower CHD risk. Also, as most cheeses are not homogenised, they still contain milk fat globule membranes. Rosqvist *et al*.^(46)^ reported that intake of milk fat enclosed by milk fat globule membranes did not impair the lipoprotein profile when compared with butter oil. When evaluating the association between SFA and CHD after excluding the contribution from cheese, intake of SFA was still associated with lower risk of CHD. The penalised spline illustrates almost the same pattern as for total SFA, but with a tendency of higher risk at higher intakes.

A systematic review and meta-analysis found that when comparing the highest *v*. lowest intake of SFA, there was no association observed between SFA intake and CHD (RR 1·03, 95 % CI 0·98, 1·07)^(18)^. Another meta-analysis of cohort studies found that the highest *v*. lowest quintile intake of SFA had a weak association with the risk of CHD (RR 1·06, 95 % CI 0·95, 1·17)^(19)^. However, the quality of the documentation was regarded as very low, and in an analysis not adjusted for cardiovascular risk factors such as serum cholesterol, there was a significantly higher risk of CHD mortality comparing the highest *v*. lowest intake of SFA (RR 1·20, 95 % CI 1·02, 1·41)^(19)^.

Our results differ from these meta-analyses and likely reflect that, in the current study population, cheese was the predominant contributor to SFA, there was a narrow range of median SFA intake in the four quartiles, and there was an inverse association between SFA and carbohydrate intake.

### Theoretical substitution analyses

Analysing the effect of one nutrient when considering the nutrients it substitutes provides another means of understanding the observed associations^(30)^. Another study suggested that reducing the intake of carbohydrates from refined grains and added sugars may produce beneficial metabolic effects that may decrease the risk of CHD^(22)^. Jakobsen *et al*.^(21)^ showed that when substituting 5 E% from SFA by carbohydrates, there was no association with fatal CHD (RR 0·96, 95 % CI 0·82, 1·13), but a statistically significant increase in the overall CHD risk (RR 1·07, 95 % CI 1·01, 1·14). When separately evaluating carbohydrates with high and low glycaemic index, only a substitution of SFA with high glycaemic index carbohydrates was associated with a higher risk of myocardial infarction (HR 1·33, 95 % CI 1·08, 1·64)^(22)^. Chen *et al*.^(47)^ evaluated the association between dairy fat and CHD in US adults and found no significant benefit of replacing dairy fat with the same energy intake from refined starch and added sugar. However, the substitution of 5 % of energy from dairy fat by carbohydrates from whole grains was associated with a significantly lower risk of CHD (RR 0·66, 95 % CI 0·62, 0·70).

The tendency for a lower risk of CHD when replacing carbohydrates with total fat and SFA may reflect a combination of the beneficial association observed between cheese consumption and CHD as well as the deleterious association between low-fibre carbohydrate intake and CHD. Total fat and SFA intake in the context of high-cheese consumption may not be generalisable to total fat and SFA intake in a low-cheese consumption context.

### Strengths and limitations

Strengths of our study include a community-based sample of men and women with a relatively long follow-up time. Only sixty participants died due to other reasons until 2009; therefore, there was minimal competing risk from other causes of death. Linkage to the CVDNOR project database assured as good as complete follow-up. Also, we had information on health status, medication use, health habits and history of CHD at baseline, enabling us to evaluate incident CHD. Further, the FFQ captured the major sources of carbohydrates and SFA expected in the current study population, and energy adjustment of the statistical models is a well-established approach for reducing the bias related to self-reported dietary data.

Theoretical substitution analyses were performed, modelling the substitution of carbohydrates with PUFA, SFA, protein and total fat. Another strength is the robustness of the results which were similar from model to model after various adjustments.

Limitations include the relatively small number of participants and events limiting stratified analyses and multivariable adjustments.

Another limitation is the lack of information on possible changes over time in diet, medications and other risk factors. Both dietary habits and food products have changed during the study period, due to the recommendations on reducing intake of SFA as the source of fat and increasing intake of whole grains as the source of carbohydrates^(31,48)^. Intake of cooking oil has tripled from the late 1990s to about 2013, and the consumption of vegetables also increased, while intake of margarine and sugar-containing food decreased according to per capita sales data^(5)^.

Blood samples were non-fasting. Since postprandial TAG remain elevated for several hours, and the Friedewald equation, used for the calculation of LDL-cholesterol, assumes fasting TAG values, LDL-cholesterol may be underestimated^(49)^. Also, most reference values for serum lipids and glucose are established on fasting blood specimen.

Further, a common problem with FFQ is systematic under- or overreporting of nutrient and energy intake, limiting the estimation of absolute intake. However, the FFQ is well suited to rank participants by dietary intake for evaluation of associations with health endpoints^(50)^. Given that FFQ are not optimal for determining absolute nutrient intake, caution is required in the interpretation of the theoretical substitution models^(51)^.

There may also be other limitations. A large proportion (75 %) of the participants reported zero intake of butter, likely reflecting underreporting. Also, we did not have extensive information on carbohydrate quality particularly for bread due to lack of historical food recipes, lack of food label details and type of carbohydrate content for the recipes from the dietary database at the end of the 1990s.

Nevertheless, when we adjusted for estimated fibre from bread, vegetables and fruit intake, total carbohydrate intake remained a statistically significant predictor of higher CHD risk. Another limitation of the current study is that the results cannot be generalisable to populations with a greater range in carbohydrate or SFA intake. In the current study, the intake of SFA varied from 4 to 25 E% and the intake of carbohydrates varied from 21 to 74 E% resulting in a narrower range of intake when we evaluated the median intake between the lowest and highest quartiles (i.e., 10–15 E% for SFA and 43–56 E% for carbohydrates), precluding our ability to generalise to lower and higher intake values. The current study can therefore not be compared with previous studies that have shown higher all-cause and cause-specific mortality associated with much lower carbohydrate intakes than our study population^(52,53)^. Finally, the available data were not appropriate for studying unsaturated fat, given the changing *trans*-fatty acid composition of unsaturated fat during the study period.

While reverse causation is a general concern in observational studies of dietary habits and disease outcomes, we noted a similar percentage of participants with a family history of CHD and similar baseline BMI values across quartiles of carbohydrate and SFA intake. Further, adjusting for family history of CHD and BMI did not alter our findings. Although we performed multivariable analyses, residual confounding may still be present.

Lastly, it is of note that at the end of follow-up, participants’ age was generally lower than the mean age of acute myocardial infarction in Norway which is 73·8 years for men and 79·1 years for women^(54)^. Thus, our findings may reflect mechanisms involved in early-onset CHD and may not necessarily be generalisable to older populations.

## Conclusion

The focus of the current study was to evaluate the importance of the interplay between SFA and total carbohydrates, including SFA sources, when evaluating the association between SFA and CHD. A high intake of carbohydrates, reflecting low-fibre and relatively higher sucrose/fructose dietary sources, and a low intake of SFA were associated with higher CHD risk in the current study population. Substituting carbohydrates with total fat was associated with lower risk. Also, SFA from cheese was associated with lower risk of CHD.

Further research evaluating potential benefits of dairy products and their nutritional constituents is warranted. Also, there is a need to clarify the relative health trade-offs between replacing carbohydrate intake with fat intake in study populations with diverse dietary habits and a wider range in carbohydrate and SFA intakes. In addition, results of our study suggest that dietary guidelines development and their communication to the public, especially regarding reductions in certain foods and nutrients, need to consider the potential health impact of alternative sources of energy.

## Supplementary material

For supplementary material accompanying this paper visit https://doi.org/10.1017/S1368980020003043. click here to view supplementary material
