# Supplementary material for: Intake of carbohydrates and SFA and risk of CHD in middle-age adults: the Hordaland Health Study (HUSK)
Source: Public Health Nutr. 2020 Sep 10;25(3):634–48. doi: 10.1017/S1368980020003043 (PMC9991815; doi:10.1017/S1368980020003043)
Supplement: Supplementary file 1 [file S1368980020003043sup001.docx]

Intake of carbohydrates and saturated fatty acids and risk of coronary heart disease in middle-age adults. The Hordaland Health Study (HUSK)

| Supplementary table 1 Associations between macronutrients and risk of incident coronary heart disease.  The Hordaland Health Study (HUSK) | | | | |
| --- | --- | --- | --- | --- |
| Intake of macronutrients, E% | Model 1  HR (95% CI)* | Model 2  HR (95% CI)^†^ | Model 3  HR (95% CI)^‡^ | Model 4  HR (95% CI)^§^ |
|  | N = 2995 | N = 2986 | N = 2883 | N = 2697 |
|  | CHD, N = 112 | CHD, N = 112 | CHD, N = 104 | CHD, N = 93 |
| Carbohydrates |  |  |  |  |
| Q1 | 1 (ref) | 1 (ref) | 1 (ref) | 1 (ref) |
| Q2 | 1∙11 (0∙63, 1∙96) | 1∙13 (0∙64, 1∙99) | 1∙26 (0∙69, 2∙29) | 1∙16 (0∙63, 2∙17) |
| Q3 | 1∙30 (0∙75, 2∙24) | 1∙32 (0∙76, 2∙29) | 1∙45 (0∙81, 2∙60) | 1∙49 (0∙82, 2∙73) |
| Q4 | 1∙63 (0∙96, 2∙76) | 1∙54 (0∙91, 2∙63) | 1∙62 (0∙92, 2∙84) | 1∙50 (0∙81, 2∙80) |
| p – trend^\|\|^ | 0∙056 | 0∙088 | 0∙082 | 0∙150 |
| Continuous, per 2E% | 1∙08 (1∙01, 1∙15) | 1∙07 (1∙00, 1∙14) | 1∙09 (1∙02, 1∙17) | 1∙09 (1∙01, 1∙18) |
|  |  |  |  |  |
| Saturated fatty acids |  |  |  |  |
| Q1 | 1 (ref) | 1 (ref) | 1 (ref) | 1 (ref) |
| Q2 | 0∙55 (0∙34, 0∙91) | 0∙55 (0∙33, 0∙91) | 0∙54 (0∙32, 0∙90) | 0∙47 (0∙27, 0∙84) |
| Q3 | 0∙55 (0∙33, 0∙92) | 0∙57 (0∙34, 0∙94) | 0∙55 (0∙32, 0∙94) | 0∙50 (0∙28, 0∙88) |
| Q4 | 0∙53 (0∙32, 0∙90) | 0∙56 (0∙33, 0∙94) | 0∙51 (0∙29, 0∙90) | 0∙47 (0∙26, 0∙84) |
| p – trend^\|\|^ | 0∙013 | 0∙021 | 0∙013 | 0∙010 |
| Continuous, per 2E% | 0∙82 (0∙70, 0∙97) | 0∙84 (0∙71, 0∙99) | 0∙80 (0∙67, 0∙95) | 0∙79 (0∙66, 0∙94) |

E%, energy percent; HR, hazard ratio; CI, confidence interval; N, number of participants; CHD, incident coronary heart disease; Q, quartile

^*^Model 1: Cox proportional hazards regression analysis adjusted for age, sex, and energy intake

^†^Model 2: Additionally adjusted for low-density lipoprotein cholesterol, high-density lipoprotein cholesterol, triglycerides, glucose (continuous), systolic blood pressure, diastolic blood pressure, and body mass index

^‡^Model 3: Additionally adjusted for hypertension, glucose intolerance, family history of infarction, statins, oral hypoglycemics (including metformin) and insulin, and anti-hypertensive medications

^§^Model 4: Additionally adjusted for smoking habits, physical activity, alcohol consumption in E%, and education

^||^P – trend, to test for linear trends across quartiles, we modelled the median intake of each quartile as a continuous variable.

| Supplementary table 2 Associations between saturated fatty acids and risk of incident coronary heart disease, stratified by smoking habits. The Hordaland Health Study (HUSK) | | | | | | |
| --- | --- | --- | --- | --- | --- | --- |
| Intake of saturated fatty acids, E% | N (CHD) | Non-smokers  HR (95% CI)* | N (CHD) | Previous smokers HR (95% CI)* | N (CHD) | Smokers  HR (95% CI)* |
| Q1 | 270 (8) | 1 (ref) | 235 (17) | 1 (ref) | 189 (17) | 1 (ref) |
| Q2 | 251 (3) | 0∙50 (0∙13, 1∙96) | 229 (5) | 0∙32 (0∙12, 0∙88) | 223 (13) | 0∙60 (0∙29, 1∙24) |
| Q3 | 241 (3) | 0∙46 (0∙12, 1∙76) | 224 (7) | 0∙45 (0∙18, 1∙10) | 251 (12) | 0∙53 (0∙25, 1∙13) |
| Q4 | 239 (1) | 0∙15 (0∙02, 1∙17) | 187 (2) | 0∙17 (0∙04, 0∙74) | 281 (17) | 0∙74 (0∙37, 1∙46) |
| p – trend^†^ |  | 0∙037 |  | 0∙005 |  | 0∙431 |
| Continuous, per 2E% | 1001 (15) | 0∙71 (0∙44, 1∙13) | 875 (31) | 0∙60 (0∙44, 0∙82) | 944 (59) | 0∙90 (0∙73, 1∙12) |

E%, energy percent; N, number of participants; CHD, incident coronary heart disease; HR, hazard ratio; CI, confidence interval; Q, quartile

*Adjusted for age, sex, energy intake and physical activity.

^†^P – trend, to test for linear trends across quartiles, we modelled the median intake of each quartile as a continuous variable.

| Supplementary table 3 Associations between carbohydrates from different food items and incident coronary heart disease.  The Hordaland Health Study (HUSK) | | | | | | | | | |
| --- | --- | --- | --- | --- | --- | --- | --- | --- | --- |
| HR (95%CI) | | | | | | | |  |  |
| Carbohydrates from food items, E% |  | Q1 (ref) | Q2 | Q3 | Q4 |  | P – trend* |  | Continuous,  per 1E% |
| Rice, pasta, flour, cereals |  |  |  |  |  |  |  |  |  |
| Model 1† |  | 1 (ref) | 0∙84 (0∙51, 1∙39) | 0∙65 (0∙37, 1∙11) | 0∙88 (0∙54, 1∙44) |  | 0∙631 |  | 1∙00 (0∙94, 1∙06) |
| Model 2‡ |  | 1 (ref) | 1∙00 (0∙59, 1∙68) | 0∙80 (0∙46, 1∙41) | 1∙13 (0∙67, 1∙91) |  | 0∙683 |  | 1∙03 (0∙97, 1∙09) |
| Bread |  |  |  |  |  |  |  |  |  |
| Model 1† |  | 1 (ref) | 1∙06 (0∙60, 1∙85) | 1∙36 (0∙80, 2∙32) | 1∙09 (0∙63, 1∙89) |  | 0∙649 |  | 1∙01 (0∙97, 1∙04) |
| Model 2‡ |  | 1 (ref) | 1∙16 (0∙65, 2∙09) | 1∙64 (0∙95, 2∙84) | 1∙23 (0∙69, 2∙18) |  | 0∙378 |  | 1∙01 (0∙98, 1∙05) |
| Soft drinks with sugar |  |  |  |  |  |  |  |  |  |
| Model 1† |  | 1 (ref) | 0∙94 (0∙54, 1∙63) | 0∙94 (0∙54, 1∙62) | 1∙13 (0∙67, 1∙90) |  | 0∙507 |  | 1∙04 (1∙00, 1∙09) |
| Model 2‡ |  | 1 (ref) | 0∙89 (0∙50, 1∙58) | 0∙89 (0∙51, 1∙55) | 1∙00 (0∙58, 1∙71) |  | 0∙860 |  | 1∙03 (0∙99, 1∙08) |
| Juice |  |  |  |  |  |  |  |  |  |
| Model 1† |  | 1 (ref) | 1.04 (0∙63, 1∙71) | 1∙00 (0∙60, 1∙67) | 0∙79 (0∙45, 1∙37) |  | 0∙328 |  | 0∙98 (0∙88, 1∙10) |
| Model 2‡ |  | 1 (ref) | 1∙30 (0∙78, 2∙17) | 1∙24 (0∙73, 2∙13) | 0∙86 (0∙47, 1∙55) |  | 0∙406 |  | 0∙97 (0∙87, 1∙09) |
| Fruit and berries |  |  |  |  |  |  |  |  |  |
| Model 1† |  | 1 (ref) | 0∙80 (0∙47, 1∙34) | 1∙01 (0∙62, 1∙66) | 0∙98 (0∙58, 1∙67) |  | 0∙882 |  | 1∙00 (0∙96, 1∙05) |
| Model 2‡ |  | 1 (ref) | 1∙06 (0∙62, 1∙82) | 1∙40 (0∙83, 2∙35) | 1∙38 (0∙78, 2∙45) |  | 0∙186 |  | 1∙04 (0∙99, 1∙09) |

E%, energy percent; Q, quartile; HR, hazard ratio; CI, confidence interval

*P – trend, to test for linear trends across quartiles, we modelled the median intake of each quartile as a continuous variable.

**†**Age, sex and energy intake adjusted Cox proportional hazards regression analysis

**‡**Adjusted in addition for physical activity and smoking habits

| Supplementary table 4 Associations between saturated fatty acids from different food items and incident coronary heart disease.  The Hordaland Health Study (HUSK) | | | | | | | | | |
| --- | --- | --- | --- | --- | --- | --- | --- | --- | --- |
| HR (95%CI) | | | | | | | |  |  |
| Saturated fat from food items, E% |  | Q1 (ref) | Q2 | Q3 | Q4 |  | P – trend* |  | Continuous,  per 1E% |
| Margarine |  |  |  |  |  |  |  |  |  |
| Model 1† |  | 1 (ref) | 0∙90 (0∙55, 1∙49) | 0∙80 (0∙47, 1∙35) | 0∙81 (0∙48, 1∙35) |  | 0∙478 |  | 1∙00 (0∙86, 1∙17) |
| Model 2‡ |  | 1 (ref) | 0∙85 (0∙51, 1∙43) | 0∙72 (0∙42, 1∙25) | 0∙68 (0∙40, 1∙16) |  | 0∙202 |  | 0∙95 (0∙81, 1∙12) |
| Butter§ |  |  |  |  |  |  |  |  |  |
| Model 1† |  | 1 (ref) | - | - | 0∙69 (0∙43, 1∙10) |  | 0∙120 |  | 0∙91 (0∙76, 1∙08) |
| Model 2‡ |  | 1 (ref) | - | - | 0∙70 (0∙43, 1∙14) |  | 0∙154 |  | 0∙90 (0∙75, 1∙07) |
| Milk and milk products |  |  |  |  |  |  |  |  |  |
| Model 1† |  | 1 (ref) | 1∙22 (0∙73, 2∙04) | 0∙76 (0∙43, 1∙34) | 1∙16 (0∙69, 1∙93) |  | 0∙861 |  | 1∙06 (0∙93, 1∙21) |
| Model 2‡ |  | 1 (ref) | 1∙60 (0∙94, 2∙74) | 0∙95 (0∙52, 1∙73) | 1∙25 (0∙73, 2∙15) |  | 0∙838 |  | 1∙02 (0∙89, 1∙17) |
| Meat |  |  |  |  |  |  |  |  |  |
| Model 1† |  | 1 (ref) | 2∙32 (1∙32, 4∙10) | 1∙69 (0∙93, 3∙07) | 1∙27 (0∙68, 2∙35) |  | 0∙927 |  | 0∙98 (0∙81, 1∙18) |
| Model 2‡ |  | 1 (ref) | 1∙94 (1∙08, 3∙47) | 1∙51 (0∙83, 2∙74) | 0∙93 (0∙49, 1∙76) |  | 0∙332 |  | 0∙89 (0∙74, 1∙08) |
| Minced meat products |  |  |  |  |  |  |  |  |  |
| Model 1† |  | 1 (ref) | 1∙24 (0∙72, 2∙14) | 1∙23 (0∙72, 2∙10) | 1∙02 (0∙59, 1∙77) |  | 0∙942 |  | 1∙01 (0∙77, 1∙31) |
| Model 2‡ |  | 1 (ref) | 1∙27 (0∙73, 2∙23) | 1∙13 (0∙64, 1∙98) | 0∙95 (0∙54, 1∙67) |  | 0∙646 |  | 0∙94 (0∙71, 1∙24) |

E%; energy percent; Q, quartile; HR, hazard ratio; CI, confidence interval

*P – trend, to test for linear trends across quartiles, we modelled the median intake of each quartile as a continuous variable.

† Cox proportional hazards regression analysis adjusted for age, sex and energy intake.

‡Adjusted in addition for physical activity and smoking habits

§Because of a large number of zero intake reporting, Cox regression compares >0 intake versus <=0 intake.
